# *Chlamydia pneumoniae* infections and development of lung cancer: systematic review

**DOI:** 10.1186/s13027-022-00425-3

**Published:** 2022-03-22

**Authors:** Nadeesha Madushani Premachandra, J. A. A. Sampath Jayaweera

**Affiliations:** Department of Microbiology, Faculty of Medicine and Allied Sciences, Rajarata University of Sri Lanka, Saliyapura, Sri Lanka

**Keywords:** *Chlamydia pneumoniae*, Lung cancer, Risk factor, Serology

## Abstract

**Background:**

*Chlamydia pneumoniae* is an obligate intracellular pathogen and is a common cause of human respiratory diseases, including pneumonia. It has been already known to have a causal relationship with some chronic diseases such as chronic obstructive pulmonary disease, asthma, and atherosclerotic cardiovascular diseases. In this review, we aim to find out the association between *C. pneumoniae* infection and lung cancer.

**Methods:**

This is a systematic review on *C. pneumoniae* infection and the development of lung cancer, based on published articles consolidated from PubMed and Google Scholar on the topic.

**Results:**

Out of 46 articles, 27 were selected and screened through the process. Twenty-four articles positively supported the hypothesis with one animal model, while 3 of them were negatively supportive. Several proposed mechanisms explain the pathogenesis with some knowledge gaps.

**Conclusion:**

Although some studies showed an association between *C. pneumoniae* infection and lung cancer, whether the *C. pneumoniae* infection is an individual risk factor for lung cancer is still debatable. And it needs further experimental studies on both humans and animals with large observational studies to better understand the association between *C. pneumoniae* infection and lung cancer.

## Introduction

*Chlamydia pneumoniae* is an obligate intracellular pathogen transmitted via aerosols. *C. pneumoniae* was discovered after two other chlamydial species that affect humans, *C. trachomatis* and *C. psittaci*, respectively [1]. It was also known as the Taiwan acute respiratory agent (TWAR). Like all the other chlamydial species, *C. pneumoniae* tends to persist in tissues. It is a common cause of human respiratory diseases and most commonly manifests as pneumoniae and bronchitis. It is responsible for 10% of community-acquired pneumoniae and 5% of bronchitis, pharyngitis, and sinusitis [1]. Respiratory infections from *C. pneumoniae* vary in different countries and populations. It is theorized that *C. pneumoniae* is associated with other acute and chronic respiratory conditions such as chronic obstructive pulmonary disease, asthma, and lung cancer [2]. It is also associated with atherosclerotic cardiovascular disease, and there are multiple lines of suggestive evidence [3, 4].

Lung cancer is one of the major health concerns with high morbidity and mortality [5]. About 6 out of 10 people with lung cancer die within one year after diagnosis of lung cancer. Lung cancer accounts for 11.6% (2,093,876 new cases) of new carcinoma cases and 18.4% of cancer deaths in 2018 [6]. The one and 5-year survival rates of lung cancer were 42% and 15%, respectively. Its dramatic rise in recent decades is mainly due to increased smoking among males and females, attributable to 90% of lung cancer cases.

Lung cancers are broadly divided into two main categories, non-small cell carcinoma (NSCC) and small cell carcinoma (small cell lung carcinoma, SCLC). NSCC accounts for 80% of the cases, while SCLC accounts for the remaining 20%. NSCC was further sub-classified into adenocarcinoma, squamous cell carcinoma, and large cell carcinoma. Adenocarcinoma is the most common type of lung cancer, accounting for more than 40% of lung cancer cases. It also accounts for 60% of NSCC cases [7]. The pathophysiology of lung cancer is not yet fully understood. However, it is hypothesized that repeated exposure to particular carcinogens may lead to dysplasia of lung epithelium, giving rise to lung cancer. Most patients will show advanced disease at their presentation, so curative surgery is an option for a minority of patients.

It is being found that chronic infection may be a predisposing factor for malignant transformation and growth, and it is attributed to over 15% of malignancies worldwide. Although there is a considerable understanding in viral oncology, the role of bacteria on oncogenesis has not been thoroughly evaluated despite having much supportive evidence to appreciate the relationship between specific bacteria and cancers. Most published research addressed several factors that will induce cancer, such as different types of toxins, some medications, smoking, and obesity. However, only a few studies deal with cancer induction via bacterial infection. Etiological association between bacteria and cancer gained the attention of the researchers after discovering the carcinogenic potential of *Helicobacter pylori.* Studying the long-term effects of bacteria has become of great importance in cancer prevention [8].

It is hypothesized that there is a correlation between *C. pneumoniae* infection and the occurrence of lung cancer, and several studies have been conducted. In this review, we aim to find out the association between *C. pneumoniae* infection and lung cancer.

## Methods

This systematic review is on *C. pneumoniae* infection and the development of lung cancer. Our goal was to find out the relationship between the *C. pneumoniae* infection and the causation of lung cancer, considering the published web data on the topic.

### Literature searches

We conducted our literature search using the web-based search engines: PubMed and Google Scholar with the language restriction of English. We used the search terms: *Chlamydia Pneumoniae* and lung cancer, *Chlamydophilia pneumoniae* and lung cancer (both genus names used for the same organism), infectious ethology for lung cancer, and complications of *Chlamydia Pneumoniae* pneumonia. The last date of the search was on the 04^th^ of August, 2021. Experimental researches based on the topics, systematic reviews, meta-analysis, retrospective case–control studies, case reports, and other journal articles relevant to this topic, published from 1997 up to 2021, were considered for this review.

### Data extraction and analysis

Using descriptive statistics, the data from different sources were synthesized, including medians and ranges. The searches and data extraction were conducted by two investigators using the same methodology. In cases of disagreements, results were reconciled through mutual discussion.

## Results

**Fig. 1:**
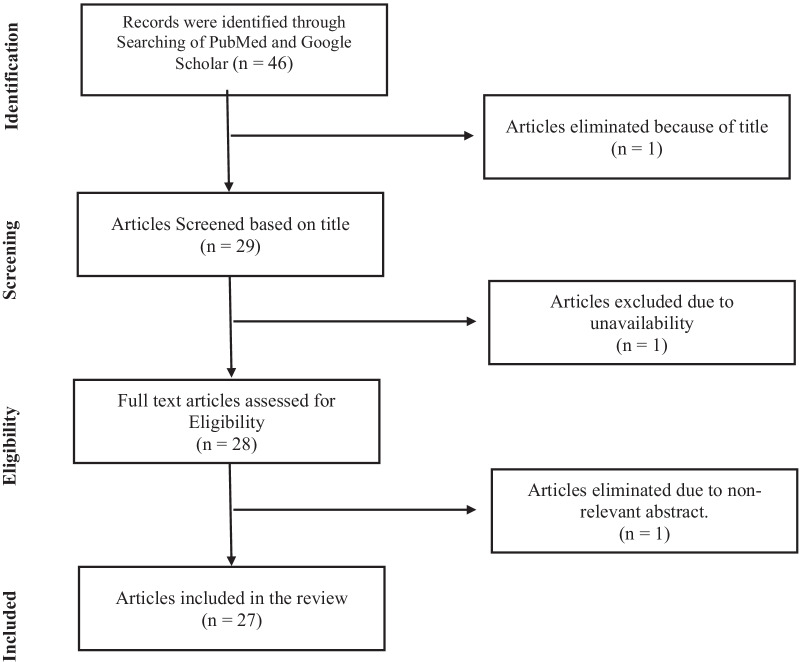
Literature review

Forty-six articles were found, and 19 articles were excluded, first considering the relevance to the title of the review than from the abstract. Among them, there was one animal study, and it was also included for the review. Selected 27 articles were screened in the process. There were 24 articles supportive of the review title, and three articles were opposive. Among them, there were three meta-analyses. We also scanned the relevant reference lists in order to identify relevant studies. In the selected case–control studies, all the cases were pathologically confirmed lung cancers, and the controls were relatively healthy people without any type of cancer. We excluded duplicate articles, presentations, textbooks, peer reviews, letters, and paper articles (Fig. 1).

### *Chlamydia pneumoniae* infection and lung cancer

The relationship between *C. pneumoniae* infection and lung cancer has been vividly studied (Table 1). In 1997, Laurila and colleagues were the ones who first postulated about the association between *C. pneumoniae* and lung carcinogenesis. The study was designed to evaluate the association between chronic *C. pneumoniae* infection and the risk of lung cancer among male smokers. Two hundred and thirty cases of smoking males with lung cancer were compared with controls, analyzed for specific antibodies and complexes for *C. pneumoniae* infection, and found that *C. pneumoniae* infection was present among 52% of cases and 45% of controls. Hence it was positively associated with lung cancer [Odds ratio (OR) 1.6; 95% confidence interval (CI) 1.0–2.3] [21].

**Table 1:** Summary of case–control studies to support the association between *Chlamydia pneumoniae* infection and lung cancer

First author, Location	Number of cases, controls	Method	Results	Conclusion
*Serology-based investigations*				
Xu X, Southeast China [9]	Cases- 449 Controls-512	All participants provided a 5 ml fasting peripheral venous blood sample for testing C. pneumoniae-specific IgG and IgA by using micro-immunofluorescence	Compared to those with no evidence of serum C. pneumoniae IgA or C. pneumoniae IgG, those with both C. pneumoniae IgG + and IgA + had 2.00 times the risk (95% CI: 1.34–3.00) of developing lung cancer	C. pneumoniae infection is potentially associated with primary lung cancer in the Chinese Han population and has combined effects with smoking, passive smoking, and a family history of cancer
Chaturv-edi AK [10]	Cases-593, Controls- 671	Assessed C. pneumoniae seropositivity and endpoint antibody titers (IgG and IgA against C. pneumoniae elementary bodies and IgG against CHSP-60). (Chlamydia heat shock protein-60 (CHSP-60) antibodies, a marker for chronic chlamydial infection.)	C. pneumoniae seropositivity by microimmunofluorescence IgG or IgA antibodies was not associated with lung cancer [odds ratio of 0.88 and 95% confidence interval (95% CI) of 0.69–1.13 for IgG; odds ratio of 0.98 and 95% CI of 0.75–1.27 for IgA]. In contrast, individuals seropositive for CHSP-60 IgG antibodies had significantly increased lung cancer risk (odds ratio, 1.30; 95% CI, 1.02–1.67), and risk increased with increasing antibody titers (P = 0.006)	CHSP-60 seropositivity and elevated antibody titers were associated with significantly increased risk for subsequent lung cancer, supporting an etiologic role for C. pneumoniae infection in lung carcinogenesis
Littman AJ [11]	508 pairs of matched cases and controls	Investigate whether IgA antibody titers to C. pneumoniae measured by the microimmunofluorescence test are associated with lung cancer risk after controlling for confounders	Individuals with antibody titers > or = 16 had 1.2 times the risk of lung cancer (95% confidence interval, 0.9–1.6) compared to those with lower titers. There was a significant trend (P = 0.007) of increasing odds ratios with increasing IgA titers primarily due to an odds ratio of 2.8 (95% confidence interval, 1.1–6.7) associated with titers > or = 256. Lung cancer risk associated with IgA titers > or = 16 was more substantial among former smokers	Future studies using precise measures of chronic C. pneumoniae status are needed to determine better the role of this organism in the etiology of lung cancer
Jackson LA [13], western Washington	Cases-143, Controls-147	Serum specimens were tested for C. pneumoniae IgG, IgM, and IgA antibodies	IgA antibody titer 216 was independently associated with risk of lung cancer among subjects < 60 years of age [odds ratio (OR), 2.67; 95% confidence interval (CI), 1.21–5.89] but not among older subjects (OR, 0.69; 95% CI, 0.34–1.43)	Additional studies, including prospective serological evaluations, are needed to assess this association's possible significance further
Laurila AL [14]	230 smoking males and matched pairs	The diagnosis of chronic infection was based on stable levels of positive specific IgA antibody (titer > or = 16) and immune complex (titer > or = 4)	Markers suggesting chronic C. pneumoniae infection were present in 52% of cases and 45% of controls and hence were positively associated with the incidence of lung cancer (OR 1.6; 95% confidence interval [CI] 1.0–2.3)	Before concluding that C. pneumoniae infection is a new independent risk factor for lung cancer, corroboration from other studies with a larger number of cases and longer follow-up is needed
Liu Z [15], China	Cases- 192 adult women, Controls-90	C. pneumoniae IgG antibodies were tested with the use of an enzyme-linked immunosorbent assay	C. pneumoniae IgG seropositivity prevalence was 61.98% of cases and 28.89% of controls (*P* < 0.05)	C. pneumoniae infection may be a risk factor for lung cancer
Kocazeybek B [16]	Cases- 123smokers, controls-123	Blood samples (5 ml) were withdrawn at the time of diagnosis and one month later. The values between IgG > / = 512 and IgA > / = 40 were set as the criteria for chronic Chlamydophila pneumoniae infections	Chlamydophila pneumoniae IgG antibody titers of > / = 512 and IgA antibody titers of > / = 40 were found at a higher rate than in the control group. This ratio was not significant for female patients. In chronic Chlamydophila pneumoniae infections, Chlamydophila pneumoniae antibody titres with values IgG > / = 512 and IgA > / = 40 were found in a total of 62 (50.4%) cases	Chronic Chlamydophila pneumoniae infections were seen statistically more often in male patients with carcinoma aged 55 years or younger. This study supports the idea that chronic Chlamydophila pneumoniae infection increases the risk of lung carcinoma
Koyi H [17]	Prospective 2 year study of 210 patients. (136 M, 74 F)	Blood specimens for Cpn serology and throat specimens for DNA analysis were taken	Both males and females had a significant prevalence of high antibody titers compared to controls	
*Molecular-based investigation*				
Xiong WM [12], China	12 matched pairs	Genomic DNA and RNA were extracted, and DNA methylation and mRNA levels were detected using the Infinium Human Methylation 450 Beadchip array and mRNA + lncRNA Human Gene Expression Microarray	According to the quantitative analysis of DNA methylation, the methylation level of the *RIPK3* promoter region was significantly different between Cpn-positive cancerous and adjacent tissues but not between Cpn-negative cancerous and adjacent tissues	Hypomethylation of the *RIPK3* promoter region increases *RIPK3* expression, leading to regulated programmed necrosis and activation of NF-κB transcription factors, which may contribute to the development and progression of Cpn-related lung cancer

A meta-analysis conducted on 12 studies using the electronic databases in 2010, which involved 2595 lung cancer cases and 2585 controls, suggested that overall, people exposed to *C. pneumoniae* infection had an OR of 1.48 (95% CI 1.32–1.67) for lung cancer risk, relative to those not exposed. This study concluded that *C. pneumoniae* infection is associated with an increased risk of lung cancer, but a higher titer may be a better predictor of lung cancer risk [22].

A study was conducted on transforming activities of *C. pneumoniae* in human mesothelial cells. This study reported *C. pneumoniae* infection to induce transformation of human mesothelial cells. Mes1 cells infected *C. pneumoniae* at a multiplicity of infection of 4 inclusion forming units/cell showed many intracellular inclusion bodies. This study suggested that *C. pneumoniae* infection might support cellular transformation leading to an increased risk of lung cancer [8]. Another meta-analysis was conducted on the association between *C. pneumoniae* infection and lung cancer in 2019. It was based on 13 study articles published from 1997 to 2013, which involved 2553 lung cancer cases and 2460 controls. This study indicated that the *C. pneumoniae* infection IgA positive rate was significantly higher among lung carcinoma patients when compared to healthy controls [2] (Table 2).

**Table 2:** Summary of studies do not support the association between *Chlamydia pneumonia* infection and lung cancer

First author	Study setting	Methods	Results	Conclusion
*Serology-based investigations*				
Smith JS [19]	Case–control study. 163 histologically confirmed cases of lung cancer and 190 controls (of whom 90 and 68 were never smokers, respectively)	C. pneumoniae IgG and IgA antibodies were measured, blinded of case–control status, using a standardized microim-immunofluorescence (MIF) assay optimized for the detection of C. pneumoniae	The prevalence of IgG positivity was 78% among cases and 74% among controls (OR 5 0.90, 95% CI: 0.52–1.57) Corresponding OR estimates were 0.65 (95% CI: 0.20–2.13) among smokers and 0.86 (95% CI: 0.43–1.73) among non-smokers	This study offers no support to the hypothesis that C. pneumoniae infection is a significant cause of lung cancer in Europe, particularly among non-smokers
Koh WP 1996–1998 [20]	Case–control study among Chinese women. Two hundred cases and 181 controls were included	Titers of IgG and IgA antibodies against C. pneumoniae were measured by indirect microimmunofluorescence (MIF) test kits	There was no association between chronic C. pneumoniae infection and lung cancer [Odds ratio (OR) 1.05, 95%confidence interval (CI) 0.61–1.80]. The null association remained when limited to non-smokers (OR 1.01, 95% CI 0.55–1.83). However, a possible association among younger subjects aged 60 years and below could not be excluded (OR 1.70, 95% CI 0.79 –3.67)	This study findings of a null association generally do not support the hypothesis that C. pneumoniae is independently associated with lung cancer among Chinese women, particularly in non-smokers
*Molecular-based investigation*				
Sessa [18]	An experimental study using lung biopsy specimens during surgery	Investigated the presence of C. pneumoniae DNA in tumor lung tissues by using real-time PCR assay. Simultaneously, tumor and healthy tissues from the same patient with primary carcinoma lung were analyzed	C. pneumoniae DNA was not detected in a single lung tumor tissue using a highly sensitive and specific real-time PCR assay based on FRET hybridization probes	This study does not support C. pneumoniae in the pathogenesis of lung cancer, suggesting that further investigations are needed to clarify other potential causative factors for the development of this malignancy

The only animal study was done using an experimental lung cancer model developed through a repeated intrathecal injection of *C. pneumoniae* into rat lungs. Among them, some of them were exposed to Benzopyrene. The antibodies (*C. pneumoniae*-IgA, -IgG, and -IgM) in serum were measured by microimmunofluorescence while *C. pneumoniae* -DNA or *C. pneumoniae* -Ag of rat lung cancer was detected using polymerase chain reaction (PCR) or enzyme linked immunosorbent assay (ELISA), respectively. Results showed the prevalence of *C. pneumoniae* infection was 72.9% in the *C. pneumoniae* group and 76.7% in the *C. pneumoniae* plus benzopyrene group, with incidences of lung cancer in the two groups of 14.6% and 44.2%, respectively (*P* = 0.001 and < 0.000 compared with normal controls) [24].

### Pathophysiology of *Chlamydia pneumoniae* infection and lung cancer

Several mechanisms are proposed to explain how *C. pneumoniae* infection could increase the risk of lung cancer. One mechanism is through mediators of inflammation. Inflammation gives rise to reactive oxygen species that may cause damage to DNA. As well as inflammation causes cell injury, resulting in consequent cell repair, increasing the rate of cell division. Given a fixed rate of DNA damage, higher cell turnover will increase the risk of a mutation conferring a selective advantage to cells, leading to cancer. There is a possible synergistic effect with *C. pneumoniae* and smoking on lung cancer pathogenesis. *C. pneumoniae* may localize more quickly in the lungs of smokers. Superoxide oxygen free radicals, tumor necrosis factor, IL-1 h, and IL-8 are produced and secreted by activated monocytes. These inflammatory mediators will cause lung tissue and DNA damage which can result in carcinogenesis. IL-8 also acts as a promoter of tumor growth for human NSCC through its angiogenic properties [25].

### Employed diagnostic methods

A case–control study investigated the role of *C. pneumoniae* infection in the pathogenesis of lung cancer, involving 449 lung cancer patients and 512 healthy controls. They were tested for *C. pneumoniae* specific IgG and IgA using micro immunofluorescence. Study results suggested that those with no evidence of serum *C. pneumoniae* IgA or *C. pneumoniae* IgG, those with both *C. pneumoniae* IgG plus and IgA plus had 2 times the risk (95% CI: 1.34–3.00) of developing lung cancer. The study concluded that *C. pneumoniae* infection is potentially associated with primary lung cancer in the Chinese Han population and has combined effects with smoking, passive smoking, and family history of cancer [9].

In an experimental study, they have investigated the association between *C. pneumoniae* IgG antibodies and the risk of lung cancer among non-smoking women. One hundred ninety-two cases of adult Chinese women and 90 healthy controls were considered in this study. Using an enzyme-linked immunosorbent assay, they were tested for *C. pneumoniae*-IgG antibodies and 61.98% of cases and 28.89% of controls were seropositive for *C. pneumoniae*-IgG [15].

A nested case–control study consisting of 593 lung cancer cases and 671 controls evaluated the relationship of *C. pneumoniae* infection and lung cancer, using traditional serological markers such as microimmunofluorescence IgG and IgA antibodies and Chlamydia heat shock protein 60 antibodies (CHSP-60), which is a marker of chronic chlamydia infection. This study showed that *C. pneumoniae* seropositivity by microimmunofluorescence IgG or IgA antibodies was not associated with lung cancer, while individuals seropositive for CHSP-60 IgG antibodies showed a significantly increased lung cancer risk [10].

A prospective 2-year study in Sweden assessed 210 (136 Males and 74 Females) patients diagnosed with lung cancer during those two years. Blood specimens for *C. pneumoniae* serology and throat specimens for *C. pneumoniae* DNA analysis by PCR were taken and analysed. Male lung cancer patients had significantly higher levels of IgG and IgA antibodies. Compared to controls, both male and female lung cancer patients had a significant prevalence of high antibody titers [17].

## Discussion

The most common form of clinical presentation of *C. pneumoniae* infection is community-acquired pneumonia (CAP). Considering the global epidemiology of pneumonia, *Streptococcus pneumoniae* is the most common pathogen causing CAP. There is an estimated prevalence of 19.3% to 34% for *S. pneumoniae* in Europe [26]. Respiratory viruses cause one-third of cases of CAP. Globally 100 million cases of viral pneumonia occur annually [27]. Intracellular pathogens such as *legionella pneumophilia, Mycoplasma pneumoniae, C. pneumoniae, C. psittaci, and Coxiella burnetii* clinically present as 'atypical' type of pneumonia. A recent review article conducted on intracellular pathogens and occurrence of pneumonia reported that the severe cases of CAP are caused by intracellular pathogens, accounting for 1% to 7% of pneumonia cases [28]. Many reports rank *C. pneumoniae* among the three most common etiologic agents of CAP, and it has an incidence ranging from 6 to 25%.

Viruses probably induce cancers through their oncogenic effects on human cells. However, the exact connection between bacteria and the oncogenic effect is less understood, and it goes the same way for the *C. pneumoniae* infection and lung cancer pathogenesis, although there are some proposed mechanisms.

According to GLOBOCAN 2020 estimates of cancer incidence and mortality, produced by the International Agency for Research on Cancer, there are 2,206,771 new lung cancer cases with a percentage of 11.4% while having a high mortality rate [29]. Considering the incidence and high mortality of lung cancer and the epidemiology of *C. pneumoniae* infection, it is essential to understand the possible causal relationship in the pathogenesis of lung cancer to implement preventive methods and encourage treatments.

All the lung cancer cases included in the studies to look for the relationship between *C. pneumoniae* infection and lung cancer were diagnosed as primary lung carcinoma after considering the clinical symptoms, examination findings, radiological findings, and confirmed with histological findings.

Different studies used various types of diagnosing methods to detect *C. pneumoniae*. Currently, there is no standard gold method to detect *C. pneumoniae* antigen levels. As serologic markers, the microimmunofluorescence test, IgA, and IgG antibody titres were widely used, while some studies used PCR for DNA analysis. Use of PCR is beneficial when there is a small amount of DNA, as it can be rapidly amplified [16]. However, the criteria used to define past infection of *C. pneumoniae* were widely varied among the studies. The lack of an exact serologic marker to identify chronic infection was a significant concern in all studies. Moreover, the reliability of those tests is still debatable. The cut-off values used in the experimental studies also varied resulting, in a comparison between studies questionable [23].

We identified nine case–control studies (9–17) and three meta-analyses [22, 25, and 30], which supported the causal association between *C. pneumoniae* and lung cancer. Only one animal study was done by using an experimental lung cancer model on rats [24]. Although these human studies reported statistically significant results using serological and DNA assays, they suggested that higher titres may be more predictable. Due to the lack of definitive serologic tests, a low number of cases, and matched controls, researchers highlighted the need for further investigations to conclude the hypothesis.

From five studies investigating the association between histologic specific lung cancer relations between *C. pneumoniae* infections, only one study reported a significant association between squamous cell cancer and small cell cancer [14]. In comparison, the two studies did not show any significant difference among histologic subtypes [13, 31]. Another study observed a strong association for squamous cell cancer but a lesser association for small cell cancer and adenocarcinoma [11]. According to a study done by Kozazeybek et al. 70 patients who had small cell lung cancer, 58.5% were positive for *C. pneumoniae*, while 28 patients who had squamous cell cancer had 50% positivity (16). However, the small number of cases does not conclude the results.

Among the relevant literature, three case–control studies do not support the causal relationship between *C. pneumoniae* infection and lung cancer [18–20]. Out of three, one study looked for *C. pneumoniae* DNA by using PCR, while they failed to detect any [18]. The other two studies measured *C. pneumoniae* IgG and IgA titres, but there was no significant difference among cases and controls [19, 20].

The causal relationship between *C. pneumoniae* infection and lung cancer would be varied when the environmental factors are considered. Hence stratified analyses are needed to be carried out by age, sex, active smoking, passive smoking, alcohol consumption, and family history of lung cancer.

## Limitations

The insufficient number of cases, selection bias, recall bias, inadequate control for confounding, and exposure misclassification are some of the main identified limitations in the studies considered for this review. Not having a standard gold test to identify chronic infection of *C. pneumoniae* and poor defining of chronic *C. pneumoniae* infection were significant limitations of all the studies. Most studies were concluded based on serology and only two studies conducted based on molecular diagnostics.

Different study designs and methodologies suggest that there may be an actual association between the occurrence of lung cancer and *C. pneumoniae* infection. Although there are some proposed mechanisms concerning causation, none of them are conclusive, and still, there are some knowledge gaps.

## Conclusion

Whether the *C. pneumoniae* infection is an independent risk factor for lung cancer is debatable, although some studies showed an association between *C. pneumoniae* infection and lung cancer. To better understand the association between *C. pneumoniae* infection and lung cancer, both experimental study designs based on animal models or large randomized controlled trials in humans and well-designed cohort studies are needed. Animal models will show a better picture of the induction and pathogenesis of lung cancer with *C. pneumoniae* infection.

## Data Availability

Please contact the author for data requests and all data relevant to the study are included in the article or uploaded as Additional information.

## Abbreviations

OR: Odds ratio
CI: Confidence interval
Ig: Immunoglobulins
PCR: Polymerase chain reaction
ESR: Erythrocyte sedimentation rate
